# Paclitaxel, but Not Cisplatin, Affects Satellite Glial Cells in Dorsal Root Ganglia of Rats with Chemotherapy-Induced Peripheral Neurotoxicity

**DOI:** 10.3390/toxics11020093

**Published:** 2023-01-19

**Authors:** Eleonora Pozzi, Elisa Ballarini, Virginia Rodriguez-Menendez, Annalisa Canta, Alessia Chiorazzi, Laura Monza, Mario Bossi, Paola Alberti, Alessio Malacrida, Cristina Meregalli, Arianna Scuteri, Guido Cavaletti, Valentina Alda Carozzi

**Affiliations:** 1 School of Medicine and Surgery, University of Milano-Bicocca, 20216 Monza, Italy; 2 NeuroMI (Milan Center for Neuroscience), 20126 Milan, Italy

**Keywords:** peripheral neurotoxicity, peripheral neuropathy, chemotherapy, paclitaxel, cisplatin, satellite glial cells, dorsal root ganglia, glial fibrillary acidic protein, gap junction

## Abstract

Chemotherapy-induced peripheral neurotoxicity is one of the most common dose-limiting toxicities of several widely used anticancer drugs such as platinum derivatives (cisplatin) and taxanes (paclitaxel). Several molecular mechanisms related to the onset of neurotoxicity have already been proposed, most of them having the sensory neurons of the dorsal root ganglia (DRG) and the peripheral nerve fibers as principal targets. In this study we explore chemotherapy-induced peripheral neurotoxicity beyond the neuronocentric view, investigating the changes induced by paclitaxel (PTX) and cisplatin (CDDP) on satellite glial cells (SGC) in the DRG and their crosstalk. Rats were chronically treated with PTX (10 mg/Kg, 1qwx4) or CDDP (2 mg/Kg 2qwx4) or respective vehicles. Morpho-functional analyses were performed to verify the features of drug-induced peripheral neurotoxicity. Qualitative and quantitative immunohistochemistry, 3D immunofluorescence, immunoblotting, and transmission electron microscopy analyses were also performed to detect alterations in SGCs and their interconnections. We demonstrated that PTX, but not CDDP, produces a strong activation of SGCs in the DRG, by altering their interconnections and their physical contact with sensory neurons. SGCs may act as principal actors in PTX-induced peripheral neurotoxicity, paving the way for the identification of new druggable targets for the treatment and prevention of chemotherapy-induced peripheral neurotoxicity.

## 1. Introduction

Chemotherapy-induced peripheral neurotoxicity ranks among the most common dose-limiting toxicities of several widely used anticancer drugs. Among them, paclitaxel (PTX) and cisplatin (CDDP) are commonly employed to treat several malignancies including ovarian, breast, lung, bladder, prostate cancers, and other carcinomas [1,2]. PTX-induced peripheral neurotoxicity (PIPN) consists of a length-dependent axonal, predominantly sensory neuropathy that usually occurs at a cumulative dose higher than 1400 mg/m^2^ [3,4,5]. CDDP induces peripheral neurotoxicity (CIPN) in a dose- and time-dependent manner, with approximately 92% of patients developing neurotoxic symptoms at cumulative doses over 500 mg/m^2^ [4]. Sensory symptoms include numbness, paresthesias, and burning pain in a symmetrical “glove-and-stocking” distribution. Although mild symptoms have been reported to resolve within several days or months after discontinuation of therapy, chronic neuropathy occurs in up to 60% of treated patients, strongly impairing their quality of life [5,6].

Until now, the molecular mechanisms underlying chemotherapy-induced peripheral neurotoxicity are still not fully understood and no effective treatment for sensory symptoms is available.

The features of neurotoxic damage depend on the chemotherapy drugs employed. PTX is an anti-tubulin agent that acts by blocking microtubule disassembling, interfering with cellular replication in cancer cells (by altering the formation of the mitotic spindle), and eventually causing their death by apoptosis. In the peripheral nervous system, several studies demonstrated that microtubule instability in peripheral nerve fibers provokes axonal degeneration and alterations in the normal nerve functioning in rodents. Differently, CDDP is a DNA-alkylating agent, able to destabilize the 3D structure of the DNA with a consequent alteration on DNA replication causing cell death. In the peripheral nervous system, several in vitro and in vivo studies performed in the 1990s demonstrated that sensory neurons of the DRG, where CDDP preferentially accumulates, are the main target of itsneurotoxicity, creating DNA-Pt adducts and inducing nucleolar structural abnormalities [7,8,9].

Peripheral sensory neurons in dorsal root ganglia (DRG) are highly susceptible to drug accumulation (including PTX and CDDP) being devoid of the blood–brain barrier and for a long time, they have been considered the only reasonable targets of investigations that led to the proposal of several pathogenetic mechanisms [10,11,12,13,14]. Nonetheless, recent data suggest that non-neuronal actors are also involved in neurotoxicity onset and pain development. In fact, DRGs do not only contain the cell bodies of primary sensory neurons but also a variety of other cell types such as a specific form of glia, called satellite glial cells (SGCs), that surround neuronal soma and play an important function in controlling the neuronal microenvironment [15,16]. In addition, SGCs are replicating cells, thus being potentially more vulnerable to the action of anti-replicating agents such as anticancer drugs.

In several inflammatory pain and axotomy models, morphological and functional alterations of SGCs as well as modification of SGCs interaction with the primary afferent neurons were reported [17,18,19,20,21,22]. It is well known that SGCs activation is commonly characterized by their proliferation and by the upregulation of the astrocyte marker glial fibrillary acid protein (GFAP). More specifically, peripheral nerve injury and inflammation can activate SGCs in rodent DRG resulting in a significant increase in the levels of GFAP, which is normally present only at a low level in perineuronal SGCs [20,22,23,24,25,26]. Moreover, it was observed that SGCs activation after an injury is usually accompanied by increased gap junction-mediated coupling between SGCs. In fact, in normal conditions, the SGCs are usually coupled only to other SGCs surrounding the same neuron while, after axonal damage, a significant increase in the coupling between SGCs enveloping different neurons was detected [27,28].

In this work, we expand the knowledge on the possible pathogenic role of SGCs in peripheral neuropathy, investigating the changes in their structure and biochemistry in well-characterized rat models of chronic PIPN and CIPN, unveiling new mechanisms of interactions between SGCs and with sensory DRG neurons.

## 2. Materials and Methods

### 2.1. Animals

Forty-eight female Wistar rats (175–200 g at the beginning of the study) were purchased from Envigo Laboratory (Udine, Italy). The animals were housed under a 12 h light/dark cycle in the animal facility-controlled rooms (maintained at 22 ± 2 °C with a relative humidity of 55 ± 10%) with ad libitum access to food and water. Their clinical conditions were monitored daily whereas their weight was recorded once or twice a week for drug dose adjustment. All experimental procedures were conducted in conformity with the institutional guidelines in compliance with national (D. L.vo 26/2014, Gazzetta Ufficiale della Repubblica Italiana, n.61, 14 March 2014) and international laws and policies (European Union directive 2010/63/UE; Guide for the Care and Use of Laboratory Animals, U.S. National Research Council, 1996). The procedures were authorized by the Italian Ministry of Health (authorization number N. 1161/2016-PR, 12 December 2016). Throughout the duration of the study, the rats were monitored daily for evidence of debilitation due to drug treatments, which is indicated by changes in their appearance (i.e., piloerection, kyphosis, mucosal dehydration, rhinorrhea), behavior (decreased grooming, eating, and drinking) and activity (decreased exploring and nesting). Any animal demonstrating evident signs of suffering or affected by a body weight decrease > 20% from the beginning of the study would be euthanized.

### 2.2. Drugs

Paclitaxel powder (PTX; LC Laboratories, Woburn, MA, USA) was dissolved in a vehicle solution composed of 10% tween 80, 10% EtOH absolute, and 80% saline solution and injected intravenously (i.v.) at the dose of 10 mg/Kg, 1 mL/Kg. Cisplatin (CDDP 1 mg/mL solution, Accord Healthcare Limited, Middlesex, UK) was injected intraperitoneally (i.p.) at the dose of 2 mg/Kg, 1 mL/Kg [29]. PTX and CDDP solutions were prepared fresh each day of drug injection.

### 2.3. Anesthesia and Euthanasia

For the recordings in the peripheral nerves, anesthesia was induced in a chamber with 3% isoflurane carried in oxygen followed by 1–1.5% isoflurane by nose cone for maintenance during the procedures. The corneal blink response and any withdrawal physical response to external stimuli were adequately suppressed. To avoid isoflurane-induced hypothermia, the body temperature was maintained at 37 ± 0.5 °C using a heating pad (Homoeothermic System, Harvard Apparatus, Holliston, MA, USA). At the end of the treatment, the rats were anesthetized with isoflurane and bled by aortic puncture.

### 2.4. Experimental Design

Rats were randomized into four groups. Twelve rats were treated with PTX 10 mg/Kg, i.v. in the tail vein, once a week for four weeks (cumulative dose 40 mg/Kg), and twelve rats were injected with CDDP 2 mg/Kg, i.p., twice a week for four weeks (cumulative dose 16 mg/Kg). The remaining twenty-four animals were divided into two groups of twelve animals each and treated with the respective vehicles’ solutions (VEH _PTX_ and VEH _CDDP_). All the animals in the study underwent neurophysiological and neuropathic pain assessments. Neurophysiological analyses were performed to assess the functionality of peripheral nerves at baseline and at the end of drug treatments; neuropathic pain was evaluated at baseline and at the end of drug treatments through behavioral tests to detect the mechanical thresholds. After in vivo evaluations, three days after the last drug injection, animals were sacrificed and employed for tissue collection. Three rats/group were used to collect DRG (L4-L6) and nerves (sciatic and caudal) for qualitative and quantitative evaluations at light and electron microscopes; three rats/group were used to harvest DRG for immunohistochemistry (IHC) in 2D. From t rats/group, DRG was collected for 3D immunofluorescence (3D-IF). From the remaining 3 rats/group, the DRG pool was harvested for the Western blot (WB) and the skin for the quantitative analysis of intraepidermal nerve fibers (IENF). For a summary of the randomization and analysis planned, see Table 1. In full respect of the reduction principle of the 3Rs, the number of animals/group (n = 12) was selected to obtain reliable results and enough biological samples to perform the analysis planned (see statistics).

### 2.5. In Vivo Evaluations for Neurotoxicity: Nerve Conduction Studies (NCS)

NCS were performed at baseline, and one day after the completion of the chemotherapy treatments. Sensory nerve conduction velocity (SCV) and sensory nerve action potential (SNAP) amplitude for both proximal caudal and digital nerves were obtained using the matrix light electromyography apparatus (Micromed, Mogliano Veneto, Italy). Stainless steel needle electrodes were used during recordings (Ambu Neuroline; Ambu, Ballerup, Denmark). During the whole procedure, rats were under deep isoflurane anesthesia while body temperature was kept constant at 37 ± 0.5 °C using a heating pad (Harvard Apparatus, Holliston, MA, USA). For the proximal caudal nerve recordings, the pair of recording electrodes were placed at 1 and 2 cm from the base of the tail and stimulating electrodes at 5 and 6 cm from the tail base; ground electrode was placed in mid-between. For the digital nerve, the recording electrodes were placed, respectively, near the ankle bone and the patellar bone, and the stimulating anode and cathode were placed at the base and at the tip of the fourth toe of the left hind limb, respectively, whereas the ground electrode was placed in the sole. Recordings filters were kept between 20 Hz and 3 KHz. Sweep was kept at 0.5 ms [30].

### 2.6. In Vivo Evaluation of Neuropathic Pain: Behavioral Test for Mechanical Thresholds

Dynamic plantar aesthesiometer test was performed to explore the mechanical thresholds of the animals, at baseline and two days after the completion of the chemotherapy regimen. Paw withdrawal threshold in response to mechanical stimulus was assessed using a dynamic plantar aesthesiometer apparatus (Ugo Basile Biological Instruments, Varese, Italy). Rats were positioned in plexiglass cages placed on a metal grid floor. After a 15 min acclimation period, a metal filament was applied to the plantar surface of the hind paw, exercising a linear increasing force ramp that reaches 50 g within 20 s. The response to the non-nociceptive mechanical stimulation was registered three times for each paw and then calculated as the average of six repeated trials (expressed in grams). The cutoff of 20 s was fixed to avoid paw damage.

### 2.7. Morphological and Morphometric Analyses on the Peripheral Nervous System

#### 2.7.1. Light and Transmission Electron Microscopy

For morphological investigations, distal caudal nerves (approximately 5 cm from the base of the tail, the point that was recorded during neurophysiological analysis) and L4-L6 DRG from three animals/group were collected, immediately immersed in 3% glutaraldehyde or 2% glutaraldehyde + 4% paraformaldehyde, respectively, post-fixed in OsO_4_ and embedded in epoxy resin for light and electron microscopy observations and quantitative analysis. For light microscopy, semithin sections of 1.5 µm thickness were prepared and stained with toluidine blue. Finally, the obtained sections were examined, and representative images were taken with a Nexcope Ne920 AUTO light microscope (TiEsseLab Srl, Milano, Italy). In order to deeply investigate the interactions between SGCs and between SGCs and sensory neurons in the DRG, ultrathin sections of 70 nm thickness of DRG from PTX and VEH _PTX_ groups were prepared and counterstained with uranyl acetate and lead citrate for ultrastructure morphological examination using a Philips CM 10 transmission electron microscope (Philips, Eindhoven, The Netherlands).

#### 2.7.2. Morphometric Analysis of DRG and Peripheral Nerves

Serial 1.5 μm sections, spaced 50 μm, were prepared for the morphometric analysis of L4-L6 DRG. Images were captured with a light microscope-incorporated camera (Nexcope Ne920 AUTO light microscope, TiEsseLab Srl, Milano, Italy) at a magnification of 20×. The somatic, nuclear, and nucleolar size of at least 200 DRG neurons/rat were manually measured from three animals/group and analyzed with a computer-assisted image analyzer (Image J software, US National Institutes of Health). The same blinded observer performed all the morphometric measurements. For morphometric analysis of distal caudal nerves, the image of one nerve section for each animal (n = 3 rats/group) was captured with a light microscope-incorporated camera (Nexcope Ne920 AUTO light microscope, TiEsseLab Srl, Milano, Italy) at a magnification of 60×. The frequency distribution of the fiber diameters and the density of myelinated fibers were calculated using an automatic image analyzer (Image-Pro Plus compiled by Immagini e Computer SNC, Milan, Italy).

#### 2.7.3. Intraepidermal Nerve Fiber Density (IENF)

To evaluate the damage of small unmyelinated peripheral nerve fibers, a parameter correlated with both neurotoxicity and neuropathic pain induced by chemotherapy, IENF density in the hind paw footpad of three animals/group was measured. Plantar glabrous skin biopsies (5 mm) from the right hind paws were fixed in 2% PLP (paraformaldehyde–lysine–sodium periodate) solution for 24 h at 4 °C and cryoprotected at −20 °C until use. Frozen samples were serially cut into 20 µm sections with a cryostat. Three sections from each footpad were randomly selected and immunostained with rabbit polyclonal anti-protein gene product 9.5 (UCHL1/PGP 9.5, Proteintech, Illinois, Rosemont, IL, USA) using a free-floating protocol. The total number of nerve fibers that cross the dermal/epidermal junction was counted from three sections/animal under light microscopy at high magnification, and the length of the epidermis was manually measured (Image J software, US National Institutes of Health). Finally, the density of IENF was obtained as a number of PGP 9.5 positive fiber/epidermal length (mm).

### 2.8. Light Microscopy for Histological Investigations

#### 2.8.1. Quantitative Immunohistochemistry for GFAP

To investigate the SGCs activation, L4-L6 DRG of three animals per group were dissected, post-fixed in 10% formalin for 3 h at RT and then paraffin embedded with HistoPro200 (HistoLine, Pantigliate, Milano, Italy). Three μm-thick serial slices were cut with a Leica RM2265 microtome (Microsystems GmbH, Wetzlar, Germany). Immunohistochemistry was performed using a rabbit polyclonal anti-glial fibrillary acid protein antibody (GFAP, Z0034, Dako Products-Agilent, Santa Clara, CA, USA). Paraffin sections were deparaffinized with xylene, rehydrated and antigens were retrieved with Proteinase K 20 ug/mL for 1 min at 37 °C. Immunolabeling was performed using an automatic Immunostainer (Autostainer 360, Epredia, Milano, Italy). Endogenous peroxidase activity was quenched by incubation in 3% H_2_O_2_ for 10 min at RT, then the slides were washed in PBS-Tween 0.5% and incubated in 5% NGS for 30 min at RT. The sections were incubated with anti-GFAP antibody (1:250 in 1% NGS) for 1 h at RT. Then, the slides were washed in PBS-Tween 0.5% and incubated with a biotinylated secondary antibody to rabbit IgG for 1 h at RT (1:200, Vector Laboratories, Peterborough, UK) followed by incubation with streptavidin-conjugated horseradish peroxidase for 1 h at RT (1:100, ABC kit Vectastain, Vector Laboratories, Peterborough, UK). The antigen–antibody complex was visualized by incubating the sections with 3.3-diaminobenzidine hydrochloride (DAB Substrate kit SK-4100, Vector Laboratories, Peterborough, UK). Negative controls were incubated only with the secondary antibody. Sections were counterstained with Haematoxylin and mounted in BioMount HM (BioOptica). Quantitative measure of GFAP-positive areas was performed for PTX and VEH _PTX_ through Image J segmentation: three DRG/group were serially sectioned at a thickness of 3 μm and immunostained for GFAP. Stitched images were acquired, spaced at 12 μm, using a scanner (Zeiss Axioscan 7, Milano, Italy) and post-processed in order to erase GFAP-positive dorsal roots by using PhotoShop software. A segmentation of post-processed images was performed using a computer-assisted image analyzer (Trainable Weka Segmentation plugin of Image J software, US National Institutes of Health) trained to recognize GFAP-positive SGCs [31]. Image J classifier was applied to all images in the batch and the results were expressed as percentage of GFAP positive area/DRG total area.

#### 2.8.2. Three-Dimensional Immunofluorescence

In order to support the quantitative immunohistochemistry and immunoblotting results, whole-mount immunofluorescence (3D-IF) of DRG was performed on PTX samples. DRG were harvested from three rats/group by keeping the animals refrigerated on ice; DRG were put into a dish containing freezing cerebrospinal fluid (CSF)-like solution (NaCl 126 mM, KCl 2.5 mM, d-glucose 10 mM, NaHCO3 26 mM, NaH2PO4 1.25 mM, CaCl2 2 mM, MgCl2 1.5 mM) and then DRG were incubated in oxygenated CSF solution with 10% collagenase (collagenase type 3, Worthington Biochemical, NJ, USA) for 1 h at 37 °C. After washing, fixation in 4% paraformaldehyde, and removal of the connective capsule, samples were ready for immunostaining, which was performed under a stereomicroscope in a 96-well plate. Primary antibodies against GFAP (1:500, Z0034, Dako Products-Agilent, Santa Clara, CA, USA), against Cx43 (1:100, sc-13558, Santa Cruz Biotechnology, CA, USA), and biotinylated-IB4 (1:250, L2140, Darmstadt, Germany) were used overnight at 4 °C. Alexa Fluor conjugated secondary antibodies (donkey anti-rabbit 555; donkey anti-mouse 488), phalloidin-Atto 647 N (1:200, Waltham, MA, USA) and Alexa Fluor conjugated Atto 565 streptavidin (1:200, Waltham, MA, USA) were incubated for 1 h at RT. Samples were mounted on “custom made” slides with a DAPI-containing mounting media and then analyzed at a confocal microscope (LSM710, Zeiss, Oberkochen, Germany).

### 2.9. Immunoblotting for Cx43

To investigate changes in the expression of gap junctions, the pool of DRG of three animals/group were collected, immediately frozen in liquid nitrogen, and stored at −80 °C until use. DRG pool from each rat was homogenized in lysis buffer (50 mM Hepes pH 7.5, 150 mM NaCl, 10% glycerol, 1% Triton X100, 1.5 mM MgCl2, 5 mM EGTA, 4 mM PMSF, 1% Aprotinin, 10 mM sodium orthovanadate and 20 mM sodium pyrophosphate) supplemented with protease and phosphatase inhibitor cocktails with a TissueLyser II (Qiagen, Milano, Italy) instrument. Protein content was quantified using the Bradford method. Proteins were then loaded in SDS-page polyacrylamide gel after chemical and thermal denaturation. After electrophoresis, proteins were transferred to nitrocellulose filters and immunoblotting analysis was performed. Briefly, antibodies against Cx43 (1:500, sc-13558, Santa Cruz Biotechnology, USA) and beta actin (1:1000, Santa Cruz Biotechnology, CA, USA) were used. After incubation with primary antibodies, the membrane was washed and then incubated with appropriate horseradish peroxidase-conjugated secondary antibodies (1:2000, anti-mouse, Chemicon, USA; anti-rabbit, PerkinElmer, MA, USA). Immunoreactive proteins were visualized using an ECL chemiluminescence system (Amersham, ImageQuant 800, Cytiva Life Sciences, MA, USA).

### 2.10. Statistics

The sample size was determined by considering the power analysis and the biological sample demand for morphological and molecular analysis described before. The power analysis (G*Power 3.1) was performed considering 80% power and 0.05 as the statistical significance, indicating minimum of 8 animals/group required, considering the SNCV the primary endpoint. The number of animals employed was definitely 12/group, in order to satisfy the biological sampling demand. Nerve conduction, behavioral test, and Western blot, IENF density data were analyzed with non-parametric t test (Mann–Whitney test). DRG and caudal nerve morphometry, and quantitative immunohistochemistry data were analyzed with unpaired t tests. A *p*-value ≤ 0.05 was set as significant. All analyses were conducted with GraphPad Prism software (v4.0).

## 3. Results

### 3.1. In Vivo Observations for PIPN and CIPN

#### 3.1.1. Drug Tolerability

The treatments with PTX and CDDP were generally well tolerated by the animals: rats continued to groom, make nests, explore their surroundings, and climb on their wire cage tops during drug treatment. Only 20% of the animals treated with CDDP showed mild piloerection, considered an initial sign of distress. The animals were weighed on drug administration days and, throughout the study, the CDDP-treated, but not PTX-treated rats, had a significant decrease in body weight compared to their respective controls (vehicle-treated), starting from the second drug administration (*p* = 0.005 day 8, *p* = 0.0014 day 11, *p* = 0.0002 day 14, *p* < 0.0001 day 17, *p* < 0.0001 day 22 vs. VEH _CDDP_; see Figure S1—supplementary material). No mortality was recorded, and no animals were euthanized prematurely during the study.

#### 3.1.2. Nerve Conduction Studies

At baseline, nerve conduction studies (NCS), together with behavioral testing were used to verify homogeneity between groups at baseline, with no significant differences between groups (See Table 2). At the end of the treatments, as expected [32,33], a significant decrease in caudal and digital SNAP amplitude and a reduction in caudal SCV were recorded in PTX-treated animals compared to VEH _PTX_, indicating the occurrence of a sensory axonal polyneuropathy (*p* < 0.0001 vs. VEH _PTX_ for caudal SNAP amplitude; *p* = 0.0016 vs. VEH _PTX_ for caudal SCV; *p* = 0.0011 vs. VEH _PTX_ for digital SNAP). By contrast, CDDP treatment did not significantly alter the NCS in both caudal and digital nerve parameters (see Table 2).

#### 3.1.3. Behavioral Tests

The mechanical threshold is a parameter of the evaluation of neuropathic pain (allodynia). As expected, mechanical allodynia, an increased sensitivity for non-nociceptive stimulations, was evident at the end of the treatment with PTX (*p* < 0.0001 vs. VEH _PTX_), as reported in Table 2. No alterations in the mechanical threshold of animals treated with CDDP were recorded.

### 3.2. Morphology and Morphometry of Peripheral Targets of Chemotherapy

**Figure 1:**
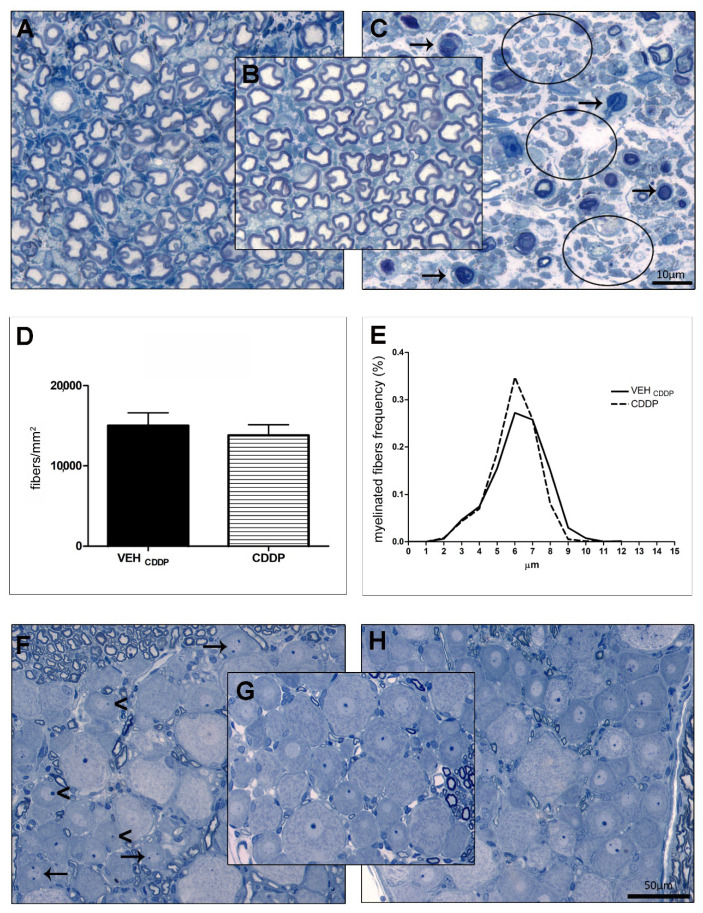
Morphological and morphometric analysis of caudal nerves and DRG. (**A**–**C**): representative images of caudal nerves at light microscopy; (**A**): cisplatin; (**B**): vehicle; (**C**): paclitaxel; (**D**,**E**): morphometric analysis of caudal nerves; (**F**–**H**): representative images of DRG at light microscopy; (**F**): cisplatin; (**G**): vehicle; (**H**): paclitaxel. Arrows and circles in (**C**) represent fiber degeneration and loss, respectively. Arrows and arrowheads in (**F**) indicate multinucleolarity and nucleolar eccentricity, respectively.

Representative sections of caudal nerves and DRG of animals treated with PTX, CDDP, and vehicles are reported in Figure 1. Table 2 reported quantitative results of morphometric investigations on DRG and epidermis.

#### 3.2.1. PIPN

Morphological and morphometric examination of caudal nerves, harvested after sacrifice at the end of treatment with PTX, confirmed the development of axonopathy after treatment: as reported in Figure 1C, an overall degeneration of myelinated fibers as well as a severe loss of fibers was evident compared to VEH (arrows and circles, respectively, in Figure 1C) in agreement with the statistically significant impaired NCS (Table 2). Morphometric analyses of caudal nerves were performed but due to the massive loss of fibers, the system was not able to affordably quantify the number and size of fibers. The quantitative analysis of small unmyelinated fibers in the skin biopsy revealed a significant decrease in IENF density (*p* < 0.01 vs. VEH _PTX_; see Table 2). Moreover, the morphological examination of L4-L6 DRG at the light microscope did not reveal any evident alteration in the cito-architecture of sensory neurons and SGCs (Figure 1H vs. VEH in Figure 1G). However, as shown in Figure 1H, an evident reduction of the extracellular space was observed between the “DRG neurons-SGCs” units. To exclude that sensory DRG neurons were enlarged we performed a morphometric examination of the somatic, nuclear, and nucleolar areas of DRG neurons that demonstrated no alterations (see Table 2).

#### 3.2.2. CIPN

At the end of treatment, we did not observe any evident change in the caudal nerve morphology of CDDP-treated animals (Figure 1A) compared to VEH (Figure 1B). However, the morphometric analysis showed, in CDDP-treated rats, a slight decrease in large fiber density (Figure 1E) and a shift on the left of the frequency distribution curve of CDDP versus VEH _CDDP_. Based on the neurophysiological results, these changes were not severe enough to perturb the NCS (Table 2). No alterations in the global fiber density were observed (Figure 1D). Moreover, the quantitative analysis of the intraepidermal fibers in the skin biopsies revealed no changes in the small unmyelinated fibers (Table 2). However, the analysis of DRG demonstrated the development of a neuronopathy: morphological examinations at the light microscope showed structural changes in the DRG sensory neurons (such as nucleolar eccentricity, arrowheads) and increased number of nucleoli (arrows, Figure 1F vs. VEH in Figure 1G). These observations were corroborated by morphometric examinations that evidenced statistically significant atrophy of DRG neurons (*p* < 0.0001 vs. VEH _CDDP_ for somatic and nucleolar areas; *p* = 0.0036 vs. VEH _CDDP_ for the nuclear area; Table 2).

### 3.3. Morphological and Morphometric Evaluations of SGCs in the DRG

#### 3.3.1. Qualitative and Quantitative Evaluations of GFAP in the DRG

**Figure 2:**
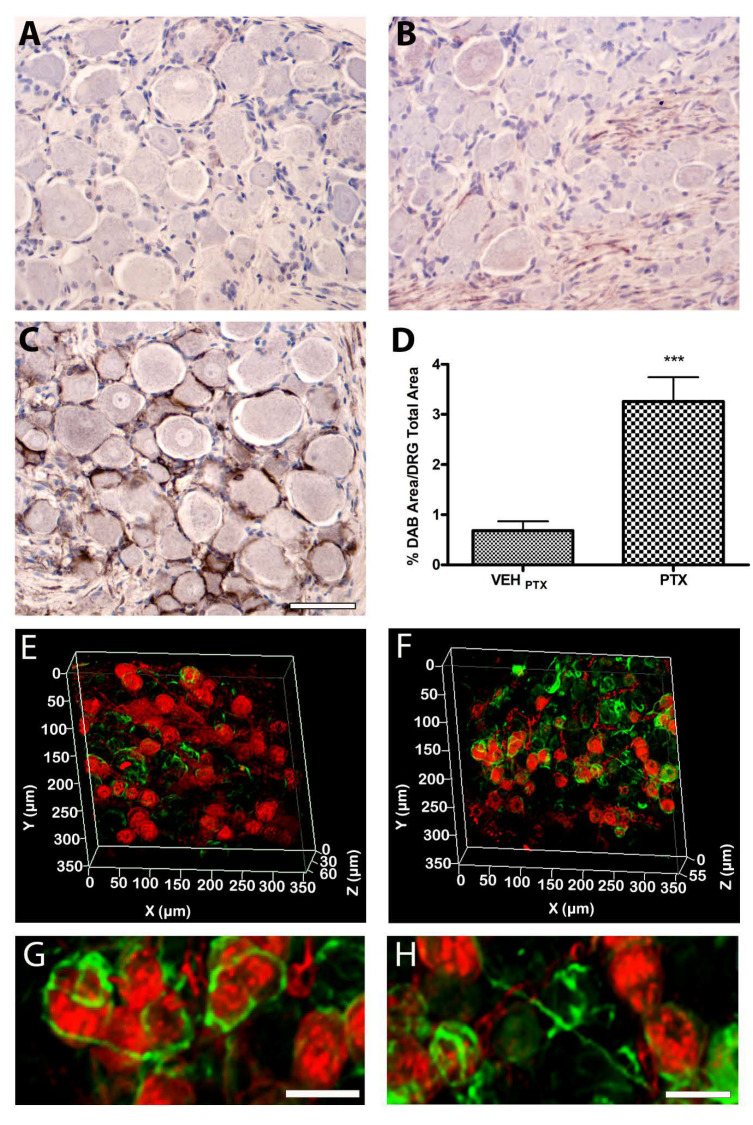
Analysis of the immunolocalization and expression of GFAP in the DRG. (**A**–**C**): immunohistochemistry analysis; (**A**): cisplatin; (**B**): vehicle; (**C**): paclitaxel; bar = 100 μm. (**D**): quantitative analysis of GFAP expression; (**E**–**H**): 3D-IF analysis (RED: IB4; GREEN: GFAP); (**E**): vehicle _PTX_; (**F**): paclitaxel; (**G**,**H**): magnifications from (**F**); bar = 25 μm. *** *p* < 0.0001.

DRG harvested from six animals/group were employed to assess differences in the immunolocalization of GFAP, a marker of glial activation widely employed in the study of CNS and PNS diseases, between PTX or CDDP-treated animals and their respective controls (vehicle-treated). We performed quantitative immunohistochemistry analysis (three animals/group) to verify the localization and quantify the GFAP immunoreactive cells in the DRG; then we performed a 3D-IF (three animals/group) to study the pattern of localization in the tissue tri-dimensional space that offer the possibility to expand the information on the distribution of GFAP. As reported in Figure 2, GFAP immunoreactivity showed a cytosolic pattern both in SGCs of chemotherapy-treated animals (Figure 2B,C) and in their controls (vehicle-treated). An evident positivity was observed also in the nerve roots, probably due to the presence of the Schwann cells. Moreover, at a qualitative observation, GFAP was strongly upregulated in SGCs of animals treated with PTX (Figure 2C), but not with CDDP (Figure 2B), compared to their controls (Figure 2A), demonstrating an SGCs activation and a possible hyperproliferation (gliosis) and/or hypertrophy in DRG of PTX animals. The quantitative analysis of immunohistochemistry, performed on DRG serial sections of PTX and VEH _PTX_ animals, confirmed a significant three-fold increase in the immunoreactive area (*p* < 0.0001 vs. VEH _PTX_, Figure 2D).

The 3D-IF, performed on three whole DRG of PTX and VEH _PTX_ animals confirmed the immunohistochemistry analysis and revealed additional information on the pattern of distribution of GFAP in the DRG: as indicated by arrows in Figure 2G and H, non-physiological connections between GFAP-positive SGCs of adjacent neurons became evident in PTX versus VEH _PTX_ (Figure 2E), a typical feature demonstrated in other pathological conditions [27], but not in normal conditions, where one or more SGCs encircle a single sensory neuron.

#### 3.3.2. Ultrastructural Evaluation of SGCs in the DRG

**Figure 3:**
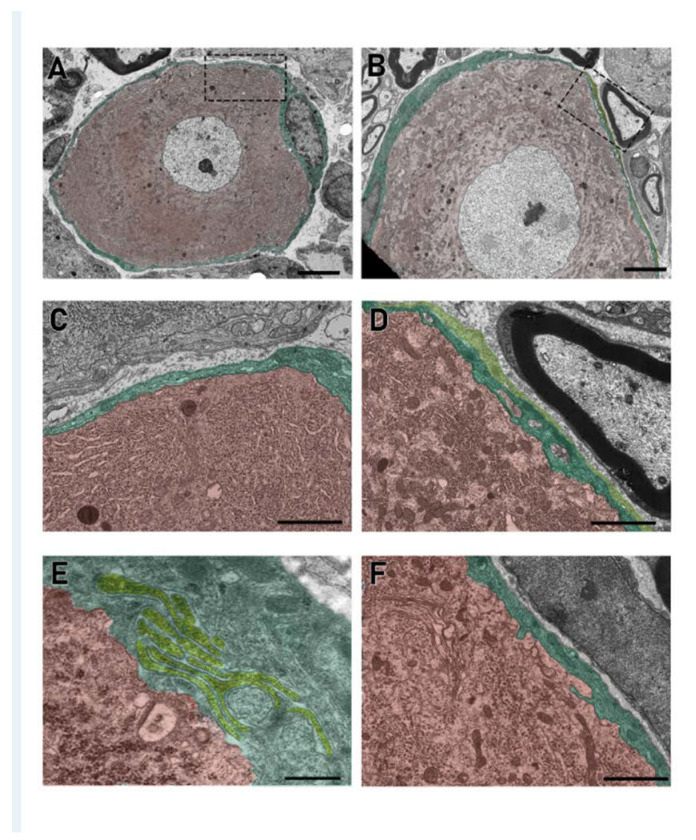
Electron microscopy analysis of the DRG cells. (**A**,**C**): vehicle; (**B**,**D**–**F**): paclitaxel. Pictures show the different interactions between SGCs and neurons. Computer-aided virtual colors help in the visualization of different SGCs (yellow and green) and neurons (red). (**D**): Two SGCs (dark and light green) wrap the same neuron (red). (**E**): An example of different SGCs membrane projections generating a complex contact internal system (light green). (**A**,**B**) bar: 5 μm; (**C**,**D**) bar: 2 μm; (**E**) bar: 0.5 μm; (**F**) bar: 2 μm.

The ultrastructural examination at the transmission electron microscope of the three DRG/animal/group was also conducted, and representative pictures are shown in Figure 3. The analysis demonstrated an increase in the intimate contact between SGCs and the associated neurons after PTX treatment compared to their vehicles. In fact, in PTX DRG, a complex and peculiar pattern of glial cytoplasmic projections was observed (Figure 3D–F) with an increased surface of interaction between SGCs (Figure 3E) and SGCs with their encircled neurons (Figure 3D,F). In addition, in the DRG of PTX-treated rats, the presence of more than one SGS wrapping a single neuron was more frequently observed (Figure 3D, dark green and light green) compared to controls. Moreover, no evident changes in the cytosolic ultrastructure of the SGCs were evident.

### 3.4. Analysis of Connections between SGCs in the DRG

Gap junctions allow a quick diffusion of ions and small molecules (<1000 Da) between connected cells. They consist of multimers of six proteins, called connexins (Cxs), that form a connexon. The connexon crosses the plasma membrane and docks with a connexon of the neighboring cell, thereby creating an extracellular gap. The level of expression and the localization of the more represented connexin in nervous system gap junctions, Cx43, have been studied in this work through Western blot, immunofluorescence, and transmission electron microscopy.

#### 3.4.1. Quantitative Analysis of Cx43 in the DRG

**Figure 4:**
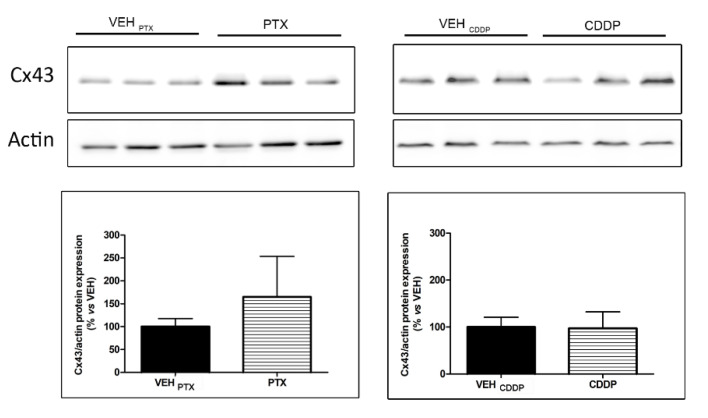
Western blot analysis and quantification for Cx43 in the DRG. VEH: vehicle; CDDP: cisplatin; PTX: paclitaxel.

The pool of DRG of three animals/group was employed to quantify, through an immunoblotting analysis, the level of Cx43 expression. The analysis demonstrated a trend towards an increase, not statistically significant, in Cx43 expression level in DRG of animals treated with PTX, but not with CDDP (Figure 4).

#### 3.4.2. Qualitative Analysis of Gap Junctions

**Figure 5:**
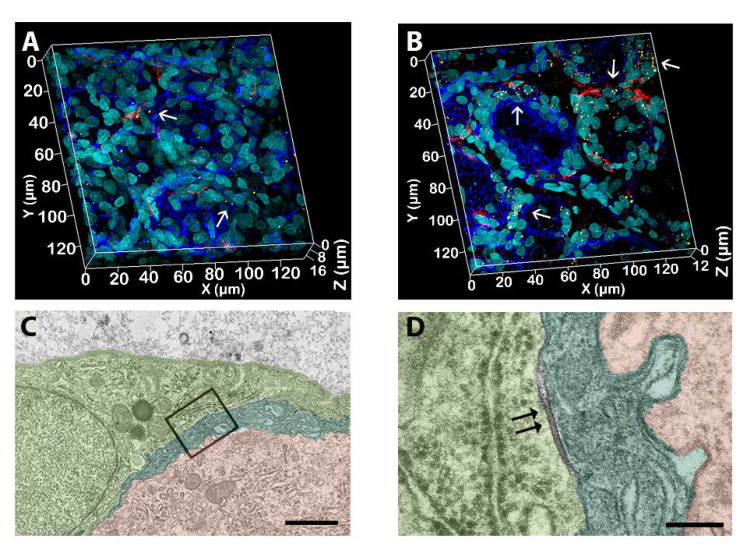
Qualitative analysis of gap junctions in the DRG. (**A**): Vehicle; (**B**–**D**): paclitaxel. Immunofluorescence shows a higher level of Cx43 (arrows, yellow dots) and GFAP (red) expression in paclitaxel-treated DRG (SGCs nuclei in cyan, phalloidin neurons in blue). Electron microscopy shows a gap junction (double arrows) between two SGCs (dark and light green) that wrap a single neuron (red) in a DRG treated with paclitaxel (**D**): magnification from (**C**). (**C**) Bar: 2 μm; (**D**) bar: 0.25 μm.

In a qualitative analysis of gap junctions, performed through 3D-IF on three DRG/animals treated with PTX and VEH _PTX_, Cx43 appeared to be distributed on the plasma membranes of SGCs (Figure 5A,B). Moreover, in PTX-treated DRG, this 3D approach revealed a more complete picture of the Cx43 distribution on SGCs surrounding neurons (blue). In fact, the immunostaining showed a pattern of discrete perineuronal localization of Cx43, forming spots of intense labeling only in PTX-treated DRG (arrows in Figure 5B), suggesting an increased concentration of gap junctions in certain points of the SGCs (nuclei in cyan) plasma membranes, and generally, an increased immunoreactivity to Cx43 in DRG of PTX-treated animals. The electron microscopy analysis permitted the visualization of the gap junctions, revealing no significant changes, induced by PTX, in their structure (Figure 5C,D).

## 4. Discussion

In this work, we performed for the first time an evaluation of the molecular changes in SGCs in the DRG after chronic exposure to chemotherapy. Chronic schedules of chemotherapy injections are, in fact, the best way to faithfully mimic the clinical conditions in which cancer patients are repeatedly treated with chemotherapy drugs and can develop, at high rates, chronic peripheral nerve damage [29,34,35]. Chemotherapy-induced peripheral neurotoxicity is dose-dependent, characterized by sensory peripheral nerve functional disturbances (i.e., paraesthesia, dysesthesia, numbness, alterations in proprioception, sensory ataxia), and in some cases also motor and autonomic symptoms may be observed. Specific symptoms are also drug-dependent: as an example, chronic injection with PTX can cause severe neuropathic pain in cancer patients, whereas CDDP, even if produces severe peripheral neuropathy, does not generally cause neuropathic pain [5,6].

A variety of potential mechanisms for chemotherapy-induced neurotoxicity has been described focusing, in the great majority of the cases, on the peripheral sensory neurons of the DRG, as the preferential target of the toxicity. In fact, unlike the central nervous system, DRG are devoid of an efficient blood–nerve barrier and are directly exposed to the toxic effects of chemotherapy drugs. Until now, the mechanisms proposed for chemotherapy-induced neurotoxicity include mitochondrial dysfunction, neuronal apoptosis, increased oxidative stress, and altered ionic transport in the somata of DRG sensory neurons as well as in their axons along peripheral nerves [34,36,37]. Moreover, considering the chemotherapy-specific mechanisms of action on cancer cells, differences in the mechanisms of neurotoxicity can be also suspected.

This study demonstrated that the chronic exposure of rats to neurotoxic chemotherapies can impact not only DRG sensory neurons and peripheral nerves, but also on SGCs. SGCs activation and changes in the interactions between different/adjacent SGCs and in the interactions between them and DRG sensory neurons were observed. However, the effect can be different for different chemotherapy drugs. PTX increased GFAP-positive areas in the DRG (suggesting a glial activation and/or hyperproliferation), increased the complexity of mutual interactions of SGC and neurons, and increased the levels of SGC interconnections via gap junctions. These results are in keeping with those of previous studies, where increased GFAP expression was seen in SGCs from different PTX-based models of neurotoxicity in rodents [38,39,40]. SGCs, in our study, displayed increased immunoreactivity to GFAP after PTX exposure, paralleled with a severe functional and structural sensory axonopathy, documented by NCS and morphological studies as well as morphometric analysis on peripheral nerve fibers. However, CDDP seemed not to have the same effect. GFAP immunoreactivity was not altered in the DRG, as well as gap junction protein Cx43. The explanation for these results can be found in the absence of a marked injury on peripheral nerves, as demonstrated by conserved neurophysiology, IENF density, and structure of peripheral nerves after CDDP exposure. Nevertheless, in our model, the establishment of peripheral neurotoxicity following CDDP administration was confirmed by the presence of a neuronopathy, evidenced by the morphometric analysis performed on DRG sensory neurons.

In our experimental conditions, CIPN consequences seemed to be milder than PIPN ones on the rat peripheral nervous system, justifying the discrepancies we observed in the SGCs activation profiles after PTX or CDDP exposures. On the other hand, the general toxicity of CDDP does not allow us to increase the cumulative dose of CDDP or to extend the time of treatment as previously demonstrated [7]. Alterations of SGCs function in peripheral neurotoxicity are not well described so far. Recent studies in models of diabetic neuropathy, oxaliplatin- or PTX-induced neuropathy, and other pain models indicated that SGCs played an essential role in the genesis of neuropathic pain [16,22,26,38,41,42,43,44,45,46]. Since SGCs completely surround and interact with the somata of DRG sensory neurons forming a distinct morphological unit, they are highly sensitive to neuronal changes. In addition, because SGCs can modulate neuronal excitability, these cells are thought to contribute to the development and maintenance of chronic neuropathic pain [21,22]. The available evidence suggests that peripheral nerve injury leads to a sensitization of sensory neurons in DRG and evokes SGCs activation. These reactions result in the release of ATP from neurons, leading to the synthesis and release of glial mediators (pro-inflammatory cytokines and chemokines) from SGCs, modulation of glutamate transporters and ion channels as well as an increased neuron–SGC–SGC–neuron coupling which in turn lead to neuronal hyperexcitability and pain [18,19,22,47,48,49]. These data are in line with our results, where functional and structural peripheral nerve alterations induced by PTX chronic administration were correlated with the SGC activation and with the occurrence of mechanical allodynia. Moreover, the correlation between the alterations of IENF and the activation of SGCs in the DRG also merits considerable attention since a sensory nerve axotomy, which can also belong to the degeneration of the IENF, could be related to the activation of SGCs [23]. In this scenario, the data we obtained in our study fit with these previous considerations: we observed a correlation between SGCs activation and alterations in their networking with neurons and with other SGCs with peripheral nerve fiber functional and structural impairment, IENF loss, and neuropathic pain. This suggests a possible indirect action of nerve terminal and nerve fiber damage on SGCs activation. If we consider the activation of glial cells a sort of “defense” mechanism adopted to rescue neurons from degeneration, this could explain the reason why, in our models, DRG sensory neurons of animals treated with PTX did not show any evident morphological alteration, whereas those of animals treated with CDDP were clearly affected. This, however, does not directly mean that PTX sensory neurons are not functionally impaired, as hypersensitized, expressing biochemical markers of hyperactivation (i.e., TRPV, Substance P), related to neuropathic pain development [50,51,52]. We can hypothesize that, depending on the extent of the axonal damage, DRG sensory neurons may undergo different electrical and biochemical changes, inducing or not SGCs activation. This could explain why, in our model of CIPN, where we observed only mild axonopathy, no signs of SGC changes were observed.

Studies aimed at investigating the mediators involved in the signaling between SGCs and sensory neurons are mandatory to clarify the molecular mechanisms of crosstalk in our experimental conditions (i.e., signaling mediated by ATP, K+, and glutamate, the main mediators of exchanges between SGCs and sensory neurons). In fact, as is well known, following the excitability of sensory neurons, ATP, K+, and glutamate released into the perineuronal space from neurons can activate the respective receptors or channels expressed on the surface of SGCs, which in turn release pro-inflammatory cytokines and ATP as well as form increased communicative connections between them through gap junctions [19,22,47]. In addition to this, the neuronal support exerted by SGCs in the DRG is also granted by optimal signaling between SGCs that is maintained by ionic exchanges (especially through intracellular Ca2+ waves and K+) through gap junctions [19,53]. Moreover, it has been hypothesized that the coupling between SGCs may contribute to and enhance peripheral pain in response to peripheral nerve injury. In fact, an increased number of gap junctions and the extent of coupling among SGCs following nerve injury are consistent evidence in several studies on pain [19,54]. Concerning the interactions of SGCs–SGCs in PIPN, we observed some connections between SGCs encircling different neurons located, in some cases, in different regions of the DRG. A similar observation was also reported by Hanani and collaborators [27], which showed that ⅓ of the SGCs of axotomized DRG had connections with SGCs of different neurons. This is a non-physiological condition, as normally one or more than one SGCs encircle a single sensory neuron, protecting and nourishing it. The increased connections after PTX-induced nerve damage may endow these cells with the ability to communicate over long distances [27]. This behavior fits with the tentative of SGCs of increasing their networking, augmenting the chances to compensate for the neuronal stress. The trend of increasing levels of Cx43 and the connections between SGCs around multiple neurons, seen in PIPN, but not in CIPN, suggested increased exchanges between SGCs, useful to neuronal homeostasis in PIPN.

Further analysis will be required also to understand the reasons why the consequences of chemotherapy treatments can be different on SGCs. Moreover, of remarkable importance is to establish whether the SGCs activation is a consequence or a participant cause of peripheral damage. In our experimental paradigm, we considered only a single time point of evaluation (end of chemotherapy treatments) and an explanation is hazardous. A further study that will consider early, mid, and late time points during the onset and development of peripheral neurotoxicity can be considered to establish the consequentiality of events.

## 5. Conclusions

In summary, in this study, we demonstrated that following PTX, but not CDDP treatment, severe axonal damage in the caudal nerve occurred together with a remarkable SGCs activation in the DRG and with changes in the interactions SGCs–SGCs and SGC-adjacent neurons.

In conclusion, the results obtained in this study contribute to shedding light on the complex combined pathogenetic mechanisms underlying peripheral toxic damage of chemotherapy and represent a novel step forward in the identification of novel “druggable” targets for the development of neuroprotective strategies against chemotherapy-induced peripheral neurotoxicity.

## Figures and Tables

**Table 1:** Experimental design. The table shows a summary of animals employed for each group, the treatments, and the analysis performed in the study. (VEH, Vehicle; PTX, paclitaxel; CDDP, cisplatin; IHC, immunohistochemistry; IENF, intraepidermal nerve fibers; IF, immunofluorescence; 3D, tridimensional.

Groups	In Vivo Analysis	Light and Electron Microscopy	IHC and IENF	3D-IF	WB
VEH _PTX_	12	3	3	3	3
PTX 10 mg/Kg 1qwx4	12	3	3	3	3
VEH _CDDP_	12	3	3	3	3
CDDP 2 mg/Kg 2qwx4	12	3	3	3	3

**Table 2:** Data recorded from the behavioral test for the mechanical threshold (dynamic test), neurophysiology, DRG morphometry, and IENFD (intraepidermal nerve fiber density) quantifications, at baseline and at end of treatments with vehicles and chemotherapies. Statistical analyses are also reported.

Tests	Parameters Evaluated		Baseline		End of Treatment		Baseline		End of Treatment		Statistical Analysis
			VEH_PTX_	PTX	VEH_PTX_	PTX	VEH_CDDP_	CDDP	VEH_CDDP_	CDDP	
**DYNAMIC TEST**	**MECHANICAL THRESHOLD**	mean	30.12	29.87	31.1	27.17 ***	29.94	30.81	31.59	32.62	*** p < 0.0001 vs VEH _PTX_ end treatment
	**(grams)**	SD	2.194	2.695	1.255	0.5398	2.349	2.059	3.335	4.808	*Non parametric t test, Mann Whitney*
		St. error	0.6615	0.7781	0.3785	0.1627	0.6781	0.5943	0.9627	1.388	
**NEUROPHYSIOLOGY**	**PROXIMAL CAUDAL NERVE AMPLITUDE**	mean	122.3	124.5	171	56.64 ***	148	142	99.35	90.09	*** p = 0.0001 vs VEH _PTX_ end treatment
	**(micronVolt)**	SD	25.22	19.57	36.04	42.16	32.12	18.1	16.03	11.31	*Non parametric t test, Mann Whitney*
		St. error	7.282	5.649	10.41	12.17	9.272	5.224	4.627	3.265	
	**PROXIMAL NERVE SENSORY VELOCITY**	mean	36.6	36.31	41	35.17 **	43.73	42.31	42.52	42.43	** p = 0.0016 vs VEH _PTX_ end treatment
	**(meters/second)**	SD	2.365	1.665	2.655	4.756	3.03	3.889	1.883	2.517	*Non parametric t test, Mann Whitney*
		St. error	0.6828	0.4807	0.7665	1.373	0.8747	1.123	0.5435	0.7267	
	**PROXIMAL DIGITAL NERVE AMPLITUDE**	mean	87.38	96.46	136.7	100.9 **	95.13	90.79	116	110.8	** p = 0.0011 vs VEH _PTX_ end treatment
	**(micronVolt)**	SD	27.8	24.43	19.32	19.65	12.85	11.24	19.96	33.52	*Non parametric t test, Mann Whitney*
		St. error	8.026	7.053	5.578	5.673	3.709	3.244	5.762	9.676	
	**PROXIMAL DIGITAL NERVE SENSORY VELOCITY**	mean	39.19	39.96	40.68	40.63	42.26	41.6	42.86	41.46	
	**(meters/second)**	SD	3.602	1.897	2.406	3.695	2.988	2.531	2.386	2.533	
		St. error	1.04	0.5477	0.6946	1.067	0.8624	0.7308	0.6887	0.7311	
**DRG MORPHOMETRY**	**SOMATIC AREA**	mean			643.3	682.4			797.7	645.6 ***	*** p < 0.0001 vs VEH_CDDP_ end treatment
	**(micron^2^)**	SD			338.2	378.2			482.1	404.9	*Unpaired t test*
		St. error			15.71	15.23			19.46	16.31	
	**NUCLEAR AREA**	mean			94.63	96.05			91.97	86.57 **	** p = 0.0036 vs VEH_CDDP_ end treatment
	**(micron^2^)**	SD			37.46	35.03			37.78	37.38	*Unpaired t test*
		St. error			1.52	1.41			1.525	1.506	
	**NUCLEOLAR AREA**	mean			7.87	8.31			9.616	7.182 ***	*** p < 0.0001 vs VEH_CDDP_ end treatment
	**(micron^2^)**	SD			5.01	4.92			5.661	4.216	*Unpaired t test*
		St. error			0.20	0.20			0.2285	0.1699	

## Data Availability

Upon publication, the raw data of this study will be available on the online archive of the University of Milan Bicocca data storage (https://board.unimib.it/collections/481e9f97-4ca9-4ffb-9050-f4c677acf379 (accessed on 1 June 2022).

## Supplementary Materials

The following supporting information can be downloaded at: https://www.mdpi.com/article/10.3390/toxics11020093/s1, Figure S1: body weight changes in drug- and vehicle-treated animals along the studies.
