# Supplementary material for: Paclitaxel, but Not Cisplatin, Affects Satellite Glial Cells in Dorsal Root Ganglia of Rats with Chemotherapy-Induced Peripheral Neurotoxicity
Source: Toxics. 2023 Jan 19;11(2):93. doi: 10.3390/toxics11020093 (PMC9961471; doi:10.3390/toxics11020093)
Supplement: Supplementary file 1 [file toxics-11-00093-s001.zip › toxics-2138830-supplementary.pdf]

toxics

Paclitaxel, But Not Cisplatin, Affects Satellite Glial Cells in Dorsal Root Ganglia of Rats with Chemotherapy-Induced Peripheral Neurotoxicity

Eleonora Pozzi [1,2,†], Elisa Ballarini [1,2,†], Virginia Rodriguez-Menendez [1,2], Annalisa Canta [1,2], Alessia Chiorazzi [1,2], Laura Monza [1,2], Mario Bossi [1,2], Paola Alberti [1,2], Alessio Malacrida [1,2], Cristina Meregalli [1,2], Arianna Scuteri [1,2], Guido Cavaletti [1,2] and Valentina Alda Carozzi [1,2,*]

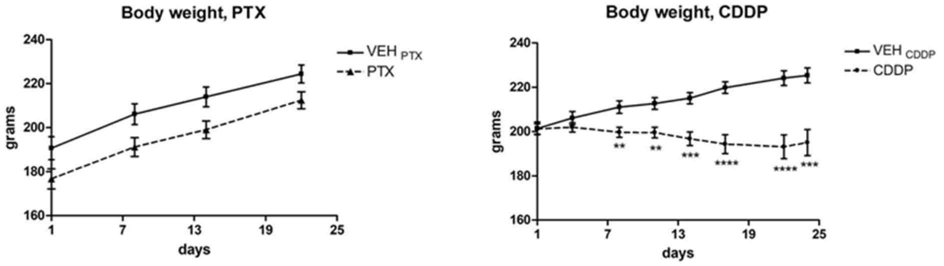

Figure S1. Body weight changes in drug- and vehicle-treated animals along the studies. ** $p < 0.01$, *** $p < 0.001$, **** $p < 0.0001$.
